# Functional genomic analysis of K^+^ related salt-responsive transporters in tolerant and sensitive genotypes of rice

**DOI:** 10.3389/fpls.2022.1089109

**Published:** 2023-01-19

**Authors:** Umme Sabrina Haque, Sabrina M. Elias, Israt Jahan, Zeba I. Seraj

**Affiliations:** ^1^ Plant Biotechnology Laboratory, Department of Biochemistry and Molecular Biology, University of Dhaka, Dhaka, Bangladesh; ^2^ Department of Life Sciences, Independent University Bangladesh, Dhaka, Bangladesh

**Keywords:** rice, CRISPR/Cas9, expression analysis, Horkuch, *OsTPKa*, *OsHAK_like*, salt-tolerant, salt-sensitive

## Abstract

**Introduction:**

Salinity is a complex environmental stress that affects the growth and production of rice worldwide. But there are some rice landraces in coastal regions that can survive in presence of highly saline conditions. An understanding of the molecular attributes contributing to the salinity tolerance of these genotypes is important for developing salt-tolerant high yielding modern genotypes to ensure food security. Therefore, we investigated the role and functional differences of two K^+^ salt-responsive transporters. These are OsTPKa or Vacuolar two-pore potassium channel and OsHAK_like or a hypothetical protein of the HAK family. These transporters were selected from previously identified QTLs from the tolerant rice landrace genotype (Horkuch) and sensitive genotype (IR29).

**Methods:**

In silico comparative sequence analysis of the promoter sequences of two these genes between Horkuch and IR29 was done. Real-Time expression of the selected genes in leaves and roots of IR29 (salt-sensitive), I-14 and I-71 (Recombinant Inbred Lines of IR29(♀)× Horkuch), Horkuch and Pokkali (salt-tolerant) under salt-stress at different time points was analyzed. For further insight, OsTPKa and OsHAK_like were chosen for loss-of-function genomic analysis in Horkuch using the CRISPR/Cas9 tool. Furthermore, OsTPKa was chosen for cloning into a sensitive variety by Gateway technology to observe the effect of gain-of-function.

**Results:**

The promoter sequences of the OsTPKa and OsHAK_like genes showed some significant differences in promoter sequences which may give a survival advantage to Horkuch under salt-stress. These two genes were also found to be overexpressed in tolerant varieties (Horkuch and Pokkali). Moreover, a coordinated expression pattern between these two genes was observed in tolerant Horkuch under salt-stress. Independently transformed plants where the expression of these genes was significantly lowered, performed poorly in physiological tests for salinity tolerance. On the other hand, positively transformed T_0_ plants with the OsTPKa gene from Horkuch consistently showed growth advantage under both control and salt stress.

**Discussion:**

The poor performance of the transgenic plants with the down-regulated genes OsTPKa and OsHAK_like under salt stress supports the assumption that OsTPKa and OsHAK_like play important roles in defending the rice landrace Horkuch against salt stress, minimizing salt injury, and maintaining plant growth. Moreover, the growth advantage provided by overexpression of the vacuolar OsTPKa K^+^ transporter, particularly under salt stress reconfirms its important role in providing salt tolerance. The QTL locus from Horkuch containing these two transporters maybe bred into commercial rice to produce high-yielding salt tolerant rice.

## 1 Introduction

Rice is one of the most important food crops globally, with 154 million hectares under cultivation and humans consuming 85 percent of the total rice produced (UNDP, 1997). However, it is considered a salt-sensitive crop with its growth and yield greatly affected under stress (Munns and Tester, 2008). Salinity is considered one of the major environmental stresses which negatively affect plant growth and productivity across the world. It is known to influence about 20% of the Earth’s land and 50% of irrigated land worldwide which includes about 30% of the rice areas (Ahmadizadeh et al., 2016). Moreover, nearly 1 million ha of coastal soil in Bangladesh, or nearly a ninth of the total cultivable area is affected by soil salinity, mostly due to sea-water intrusion (Jahiruddin and Satter, 2010). However, there are some salt-tolerant rice landraces, like Horkuch, endemic to the coastal region of Bangladesh, which are likely to harbor novel sources of salt tolerance, due to their allelic diversity, that can complement known salt tolerance genes (Razzaque et al., 2019). Understanding the factors that can provide salinity tolerance such as ensuring efficient ion homeostasis under stress to maintain their productivity, would provide the resources to develop stress-tolerant high yielding crops.

Rice is mostly sensitive at the early seedling and reproductive stages. Excess salt causes both osmotic stress and later on ionic toxicity in rice plants. Ionic stress causes chlorosis and necrosis which either impairs growth and development and/or accelerates senescence. Increased accumulation of Na^+^ in the cytoplasm also adversely affects the physiology of rice plants, such as hindering the net photosynthesis (Pn), degradation of photosynthetic pigment, stomatal conductance (Gs) and transpiration rate (Tr) (Hussain et al., 2017). These deleterious effects are not just about an increase in Na^+^; there is a consequential inhibition of K^+^ uptake because of their antagonistic effect. This event is more pronounced in salt-sensitive than tolerant plant genotypes (Wu et al., 2018). The effects of a high Na^+^/K^+^ ratio unavoidably leads to growth retardation, metabolic disruption, and even cell death, as K^+^ has a vital role in enzyme activation, osmotic adjustment, turgor generation, cell expansion, regulation of membrane electric potential, and pH homeostasis (Sharma et al., 2013). It is also a major factor in resistance to drought, salinity, and fungal diseases (Amtmann et al., 2008). However, there are some stress-tolerant cultivars in which several catalytic, DNA binding, transcription regulator activities, and metabolic pathways have been reported to be enriched (Yamaguchi-Shinozaki and Shinozaki, 2006) which can help the plant to have natural adaptation responses at physiological, molecular, and cellular levels to tolerate salinity stress.

The farmer popular rice landrace, Horkuch was previously characterized as salt-tolerant at the seedling and reproductive stages (Lisa et al., 2011). Functional enrichment for signal transduction, DNA-dependent regulation, and transport activities under stress conditions are assumed to complement salt tolerance in this landrace (Razzaque et al., 2019). In a previous study of identifying genetic loci for salinity tolerance of Horkuch, at two sensitive developmental stages, 14 QTLs for 9 traits were identified. Among them, 2 QTLs for total potassium (TK) were found to differ significantly among the Horkuch (tolerant donor) and IR29 (sensitive) at the seedling stage (Haque et al., 2022). Both the QTL intervals for TK were enriched with various transmembrane transporters including potassium ion transmembrane transporters. Vacuolar Two pore Potassium channel (*OsTPKa*) and HAK type transporter (*OsHAK_like*) reside in that QTL region (Haque et al., 2022).

Both the TPK and HAK potassium transporters are very important for K^+^ homeostasis. The TPK potassium channels mainly reside in the vacuolar membrane and are the gateway for K^+^ exchange between the vacuole and cytoplasm as and when necessary. These highly K^+^ selective TPK channels are likely involved in plant stress and developmental processes (Dabravolski and Isayenkov, 2022). The rice TPKa isoform is located in the tonoplast of the main lytic vacuole, while the TPKb is located in small vacuoles (Isayenkov et al., 2011a). The C-terminals of these isoforms determine their location. The TPK channels have the GYGD motif responsible for K^+^ selectivity, as well as Ca^2+^ binding EF and 14-3-3 domains which may play regulatory roles (Isayenkov et al., 2011b). A third rice isoform TPKc has not been well characterized as yet. The constitutive overexpression of the TPKb isoform has been reported to confer osmotic and drought tolerance to rice and lower sensitivity to K^+^ deficiency (Ahmad et al., 2016). A family of 27 HAK (often referred to as KT/HAK/KUP) transporters, most of which are located on the plasma membrane, have been reported in rice, and have been classified into 4 clusters based on their amino acid sequences (Gupta et al., 2008; Okada et al., 2018). Cluster I transporters function in high affinity K^+^ uptake, while those in cluster II are low affinity transporters. The function of Cluster III and IV transporters are not well-characterized so far.

The salt tolerance in this endemic rice landrace is regulated by myriad correlated genes and complex mechanisms. Despite progress in understanding the molecular signaling and mechanisms of salt tolerance, much remains to be learned from the natural and adaptive salt tolerance observed in landraces. Being an essential nutrient for plants, K^+^ improves the growth, development and yield of crops. K^+^ can also promote growth under stress conditions through different defense responses. For example, the reactive oxygen species generated due to the osmotic stress in excess salinity, can be ameliorated *via* K^+^ application. Also, K^+^ helps in strengthening antioxidant enzyme activities, thus improving nitrogen use efficiency. Overall maintenance of K^+^ homeostasis enhances a plant’s salt tolerance by aiding in sustainable crop yield (Kumari et al., 2021). High Na^+^ concentration in cytosol hampers cell metabolism, e.g., photosynthetic activities. Replacing K^+^ as vacuolar osmoticum is a defense mechanism for the cell under excessive Na^+^ load. K^+^ is involved in electrical neutralization of ions and macromolecules, pH homeostasis, regeneration of cell osmotic pressure, increase in leaf area, activation of enzymes, stomatal movement and tropism and translocation of ions, e.g. Na^+^, etc. Hence identifying potentially salt-responsive transporters and ion channels from the tolerant landrace Horkuch could provide a source of diverse genes for a comparative study with the existing pool of membrane transport proteins and for creating a novel artificial transporter. With a view to this, the study focuses on functional genomics analysis of some differentially expressed genes related to salt-responsive K^+^ channel *OsTPKa* (LOC_Os03g54100) and putative K^+^ transporter *OsHAK_like* (LOC_Os03g55370) from Horkuch.

In this study, we have carried out *in silico* comparative structural and functional analysis, differential gene expression, and loss and gain of function-based analysis of candidate transporter genes between tolerant Horkuch and sensitive IR29 to elucidate their function and likely mechanism in providing salt tolerance. If the function of these genes is proven, they can be used for selective breeding and gene editing to generate a high-yielding tolerant variety. Moreover, to have a cumulative effect, those genes can be recombined in a single variety to provide enhanced tolerance.

## 2 Methods and materials

### 2.1 Plant material

Five varieties: IR29, Horkuch, I-14, I-71 and Pokkali were selected based on their functional response towards salinity. Horkuch from the coastal region (Lisa et al., 2011) and Pokkali have shown tolerance to salt condition (Xie et al., 2000). IR29 is a modern rice variety from IRRI which is highly susceptible to salinity. I-14 and I-71 are Recombinant Inbred Lines of the F_7_ generation from the reciprocal crosses between IR29 and Horkuch (Haque et al., 2022). I-14 behaves more like Horkuch in terms of maintaining K^+^/Na^+^ homeostasis as Horkuch is the donor plant of the Potassium-QTL on chromosome 3 for I-14. On the contrary, in most cases I-71 behaves more like IR29 as IR29 is the donor plant of Potassium-QTL for I-71. BRRI dhan28 (http://dhcrop.bsmrau.net/rice-variety-brri-dhan-28/), a salt sensitive high-yielding modern variety of rice was used to overexpress the *TPKa* gene. BRRI dhan28 was used for transformation because it is a commercially popular variety liked by farmers. Moreover, it is genotypically very similar to IR29 (Rahman et al., 2019).

### 2.2 *In silico* analysis of candidate genes

As the genome of Horkuch and IR29 is unannotated, the gene sequences are not available in rice databases. To extract the sequence from raw data of Horkuch and IR29 genome, (Unpublished work, Zeba I. Seraj) local BLAST was performed. In the BLAST program, the reference sequence of *Oryza sativa* Nipponbare was used as a reference to detect the chromosomal location of specific sequences of Horkuch and IR29. For the extraction of the sequences and the CDS (coding sequence), 5′ UTRs (5′-untranslated regions), 3′ UTRs (3′-untranslated regions) of candidate genes from salt-tolerant Horkuch and salt-sensitive IR29 transcriptome was extracted. Expasy’s translate tool (https://web.expasy.org/translate/) was used to translate and predict amino acid sequences of the genes. To find out the functional differences of candidate protein between Horkuch and IR29, Provean (http://provean.jcvi.org/seq_submit.php) and TMHMM (http://www.cbs.dtu.dk/services/TMHMM/) and for detection of putative motif in promoter regions of candidate gene PLACE (http://www.dna.affrc.go.jp/PLACE/) was used.

### 2.3 RNA extraction, cDNA synthesis and quantitative RT-PCR

Total RNA was extracted from leaf and root of 18-day old seedlings after 0, 12, 24, 36, 48 and 72 h of 150 mM NaCl stress using TRIZOL reagent (Invitrogen, Carlsbad, CA, USA). Total RNA concentration and purity was determined using a Nanodrop ND1000 spectrophotometer (Thermo SCIENTIFIC). The cDNA was synthesized from 1.5μg of total extracted RNA using the Invitrogen Superscript III reverse transcription (RT)-PCR following the manufacturer’s protocol (Invitrogen, USA) in a final volume of 20μl. The final cDNA products of 1500-1700 ng/µl concentration were diluted 4-fold prior to use in real-time PCR.

Primers for qRT-PCR were designed using Primer3web program (http://bioinfo.ut.ee/primer3). The primers are listed in **Supplementary Table 1**. In quantitative Real-Time PCR analysis, a total of 3 replicates for all the samples of both leaf and root were used. As a control for genomic DNA contamination, an equivalent amount of total RNA without reverse transcription was tested for each sample per gene. Quantitative PCR was done by a 6μl reaction using SYBR Green (Bio-Rad, USA) with gene-specific primers in CFX96 TM Real-Time PCR detection system (Bio-Rad, USA). Using the comparative cycle threshold method, the relative abundance of transcripts was calculated. Elongation Factor- α (EF- α) was used as the normalization control. The thermal profile of the reaction was 95°C for 3 min activation and denaturation, followed by 41 cycles of 95°C for 10 sec, and 62°C for 1 min. At, 62°C step the amplification was determined by SYBR Green. Finally, a dissociation curve was generated by increasing temperature starting from 65 to 95°C to create the melting temperature curve for amplicon validation. For statistical analysis mean variables of gene expression between different genotypes and at different time points were compared with the two-way analysis of variance (ANOVA) followed by Tukey’s HSD *post hoc* test. The p < 0.05 and p < 0.01 were considered as significant and highly significant change with respect to control.

### 2.4 Construction of the sgRNA-Cas9 expression vector

Two single sgRNA were designed against *OsTPKa* (LOC_Os03g54100) and *OsHAK_like* (LOC_Os03g55370) using CRISPR-P web base resource (http://crispr.hzau.edu.cn/CRISPR2/) (Xie et al., 2014). The PAM sequence was set to be 5´-NGG-3´ (SpCas9 from Streptococcus pyogenes). The guide sequence length was defined to be 20 nucleotides and the locus tag of the above-mentioned genes were also provided. In the preliminary step, the sgRNA sequences (**Supplementary Table 2**) with an on-score of greater than 0.05, a GC content between (30-80%) and a lower off-target were chosen.

*pRGEB32* plant binary expression vector was obtained from Addgene (Addgene plasmid # 63142) (Xie et al., 2015) which contains rice snoRNA U3 (Os U3p) promoter and UBI promoter (UBIp) for simultaneous sgRNA and Cas9 expression respectively. The 20 bp long multiple cloning site was removed by *BsaI* restriction enzyme and the designed sgRNA was inserted into the vector by restriction, digestion and ligation reaction according to provider’s instructions (Xie et al., 2014). The *sgRNA_pRGEB32* constructs were transformed into *E.coli* using heat shock transformation.

### 2.5 Molecular confirmation of sgRNA constructs by PCR and sequencing

Specific sgRNA inserted plasmids were isolated from the *E.coli* colonies using Promega plasmid isolation kit protocol. Then the plasmids were confirmed by PCR reaction using Primer sets 4, 5, 6, 7 (**Supplementary Table 1**). PCR analyses were carried out in a 25 μl reaction mixture containing 100 ng of plant DNA, 100 μM of each dNTP, 2.4 ng each of primers, 1 unit of Taq DNA polymerase (Invitrogen, USA), 1.5 mM MgCl_2_, 2.4% DMSO and 1 × PCR Buffer(-MgCl_2_) (Invitrogen, USA). The optimized reaction was: Initial denaturation at 95°C for 5 min, 35 cycles of denaturation at 95°C for 1 min, annealing at 62.5°C for 1 min and extension at 72°C for 1 min following a final extension at 72°C for 10 min. The PCR-positive colonies were sequenced using primer sets-8 (**Supplementary Table 1**) by First Base DNA sequencing services from Malaysia.

### 2.6 *Agrobacterium*-mediated *in planta* transformation

The *pRGEB32_sgRNA* vector was transformed into *Agrobacterium tumefaciens* strain LBA4404 by applying standard protocols (Sambrook et al., 1989). The insertions were also confirmed by gene specific PCR. Transformed *Agrobacterium* strain (LBA4404) containing specific sgRNA construct was cultured and prepared for *in planta* transformation by following the standard protocol discussed in Parvin et al. (2015) and adapted according to Ahmed et al. (2018). Following the protocols mentioned by Amin et al. (2012), mature seeds of Horkuch were sterilized and two-days-old, germinated seeds were used for *Agrobacterium* inoculation. During the transformation events, each time embryonic apical meristem of germinated seeds of Horkuch were infected with the transformed *Agrobacterium* strain. Infected seeds were then incubated in the dark at 28°C for 6–7 days. Later, the seedlings were treated with carbenicillin solution (250 mg/l) for 1 hour to kill and remove the remnants of *Agrobacterium*. After that, seedlings were kept in light for 16 hours and in dark for 8 hours at 28°C.

When the seedlings turned green, these were transferred to hydroponic solution. After 2-3 days, the hydroponic pots were transferred to a net-house. When the seedlings were 15-20 days old, they were transferred to soil and allowed to grow and set seeds.

### 2.7 *Hygromycin* resistance test

For the *hygromycin* resistant assay, the plant leaves were cut into 3-6 pieces with a length of about 2 cm at the heading stage and then immediately soaked in Petri dish containing selected solution (20 mg/L *hygromycin*). The plates were incubated under both dark and light condition (16h day/8 h night) at 25± 2°C.

### 2.8 Leaf disc senescence assay

To evaluate the stress tolerance potential of a plant, leaf disc senescence (LDS) assay was done after the maturation of plants. Flag leaf of around 1.0-cm dimension were cut from fully developed T_0_ transgenic lines and wild type (WT) plants and then allowed to float in a 20 mL of 150 mM NaCl solution (Sanan-Mishra et al., 2005) for 3-7 days at 25°C temperature. There was no added K^+^ or K^+^ adjustment.

### 2.9 Seed germination test

To compare the germination level under salinity stress, both the non-transformed and *OsTPKa*_*sgRNA* and *OsHAK_like_sgRNA* transformed Horkuch seeds were subjected to 100 mM salt-stress at germination. After 8 days, shoot and root length were measured and compared.

### 2.10 Salt-stress screening at the seedling stage

The salt-stress screening of non-transformed and sgRNA transformed Horkuch was done according to the protocol described by Amin et al. (2012). 50mM salt-stress was applied to the 14 days old seedling with a gradual increment of 50 mM up to 150 mM stress. After 9 days of salt application the samples were collected and different parameters like electrolyte leakage, H_2_O_2_ concentration, chlorophyll content, root, shoot length, and weight were measured. These protocols were followed for salt stress screening of the overexpressed *TPKa* transgenic lines as well with gradual increment of 30 mM up to 120 mM salt stress.

#### 2.10.1 Measurement of relative electrolyte leakage

0.1g of leaf sample were taken in a 25mL falcon tube in which there was deionized water and kept in a shaker for 2 hours. After that, the initial electrical conductivity (C1) was measured using a conductivity detector. Then the leaf segments in deionized water were autoclaved, cooled down to room temperature and final electrical conductivity (C2) was measured. The values of C1 and C2 were used to calculate relative electrolyte leakage.

#### 2.10.2 Measurement of the chlorophyll concentration

0.1g leaf sample was weighed from both wild type and transgenic lines and was kept in a bottle submerged in 12mL 80% acetone solution. The leaf sample containing bottles were kept in dark for 72 hours, followed by absorbance measurements at 663nm and 645nm. The chlorophyll content was measured using the equation, A=ECd, (A is proportional to C, as E and d is constant) where A = observed absorbance; C = chlorophyll concentration (mg/mL); d = distance of the light path (= 1 cm); E = a proportionality constant (extinction Co-efficient) (=36 mL/cm).

#### 2.10.3 Measurement of H_2_O_2_ level

0.3gm leaf sample from wild type and transgenic lines were collected and crushed with liquid nitrogen, followed by adding 5 mL of 0.1% (w/v) TCA. This mixture was then centrifuged at 12000rpm for 15 minutes at room temperature. Then 3mL supernatant was collected in screw cap tube and 0.5 mL of 1 M potassium phosphate buffer (pH 7.0) and 1 mL of potassium iodide (1 M) were added. Finally, the absorbance of the mixture was taken at 390nm using 1 mL of 0.1% (w/v) TCA and 1 mL of potassium iodide as blank control. The amount of H_2_O_2_ was calculated using the equation: H_2_O_2_ (µmole.g^-1^FW) = 1+ (227.8*OD_390_).

#### 2.10.4 Measurement of Na^+^ and K^+^ content

Shoot and root from seedling of transgenic and wild type were washed and dried in oven. Dried leaves and roots were grinded and analyzed by a flame photometer after 48 hours extraction with 1N HCL according to the protocol of Yoshida (1976).

### 2.11 Construction of *pENTR-D-TOPO-OsTPKa*

Total RNA was isolated from 16 days-old salt (NaCl) stressed (100 mM) *O. sativa* cv. Horkuch using Trizol and first strand cDNA was synthesized using oligodT following manufacturer’s protocol (Invitrogen, USA). PCR was performed to amplify the target sequences of *OsTPKa.* Primer set 9 were designed for the whole fragments (1.0 kb) of *OsTPKa* (**Supplementary Table 1**). The forward primers were designed adding *CACC* overhang at the 5′ end to ensure correct orientation while cloning into the entry vector (*pENTR-D-TOPO*). PCR reactions for the target fragment was performed at 95°C for 5 min, 35 cycles of 1 min at 95°C, 30 s at 61°C, 2 min at 72°C followed by a final extension of 10 min at 72°C. The resulting fragments were gel purified using Qiagen Gel Purification system and later cloned to *pENTR-D-TOPO* vector following the manufacturer’s protocol (Invitrogen, USA). Positive clones were initially selected based on the lysate PCR confirmation from *kanamycin* resistant clones of transformed *E. coli* cells. Plasmid isolation was done from confirmed colonies using Promega plasmid isolation kit (Promega, USA). Isolated plasmids were digested with *Pst*I to check the insertion of the desired fragments into the entry vector. Then, digestion-positive colonies were sequenced with M13 forward and reverse primers Primer set 12 by First Base DNA sequencing services from Malaysia. Clones with the confirmed sequences were then recombined with the destination vector (*pH7WG2.0*) by LR reaction (Invitrogen, USA) (Karimi et al., 2002). Positive clones were selected by gene specific PCR and restriction digestion. The constructs were then transformed into *Agrobacterium* and used to infect the embryonic apical meristem of germinated seeds of salt-sensitive variety BRRI dhan28 using *in planta* transformation method as described above.

## 3 Results

### 3.1 *OsTPKa* and *OsHAK_like* sequences showed structural and functional differences in tolerant and sensitive variety

No sequence variation in CDS (Coding Sequence) and amino acid sequence was found but a significant variation was observed for both genes in the promoter region between the tolerant and sensitive genotypes which may result in variation in the expression of these genes.

For functional characterization of the differences in the promoter region, promoter sequences isolated from salt tolerant Horkuch and sensitive IR29 were scanned using the PLACE (Higo et al., 1999) programs. For *OsTPKa*, differences in the pattern of motifs between these two varieties were observed, among which auxin, jasmonate, abscisic acid, water stress, and dehydration responsiveness motifs were found in higher amounts in tolerant Horkuch whereas a negative regulatory element for the inducible expression of WRKY18 was present in sensitive IR29. Similarly, for *OsHAK_like* promoter, auxin and dehydration responsive motifs were more abundant in Horkuch. Additionally, tissue-specific expression related motifs and gibberellin-responsive motif was found to be present in Horkuch but absent in IR29. As shown below, salt stress induces increased expression of *OsTPKa* and *OsHAK_like* in the root of the tolerant genotype.

### 3.2 Tissue-specific expression analysis

**Figure 1:**
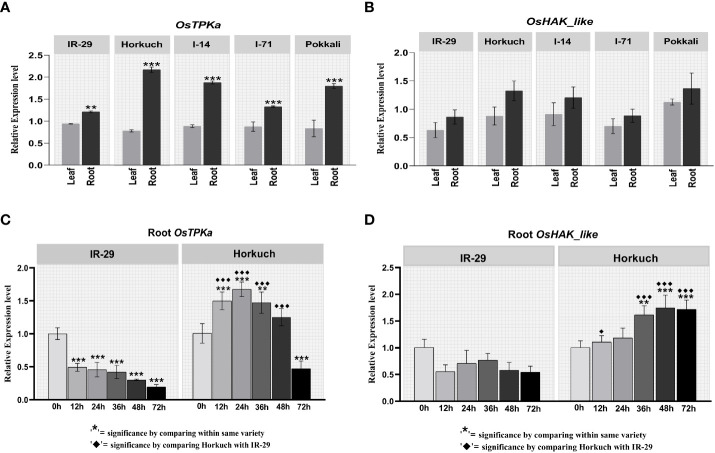
Differential expression analysis of *OsTPKa* and *OsHAK_like* by Real-Time quantitative PCR. **(A, B)** tissue specific expression analysis in IR29, Horkuch, I-14, I-71 and Pokkali at 24 hour under 150 mM salt-stress; **(C, D)** Salt-responsive genotype specific expression analysis in root of IR29 and Horkuch at 12 hour, 24 hour, 36 hour, 48 hour and 72 hour under 150 mM stress condition.

The expression pattern of *OsTPKa* and *OsHAK_like* was analyzed in leaf and root tissue of IR29, Horkuch, I-14, I-71 and Pokkali at 24 hours under 150 mM salt stress condition. Both genes showed higher expression in the root compared to the leaf region in all the genotypes. In addition, the expression in root was found to be much higher in salt-tolerant Horkuch, Pokkali and I-14 compared to IR29 under salt-stress (**Figures 1A, B**)

For a more detailed understanding of the differences in the expression pattern of *OsTPKa* and *OsHAK_like* gene between tolerant and sensitive genotypes, differential gene expression analysis was carried out at different times in both leaf and root of IR29 and Horkuch in 150 mM salt stress.

In leaf samples, for both *OsTPKa* and *OsHAK_like* genes no significant difference was observed between tolerant Horkuch and sensitive IR29. However, in the roots of IR29, *OsTPKa* showed a significant reduction in expression level at various time points under salt stress compared to non-stress, whereas a significant escalation in the expression was observed in Horkuch (**Figure 1C**). Similarly, *OsHAK_like* showed a reduction in expression level at all the observed time points under salt stress conditions compared to control in IR29. But for Horkuch, there was a gradual increase in expression compared to control at all time points under salt stress (**Figure 1D**).

### 3.3 *OsTPKa* and *OsHAK_like* showed coordinated expression pattern in tolerant genotype

**Figure 2:**
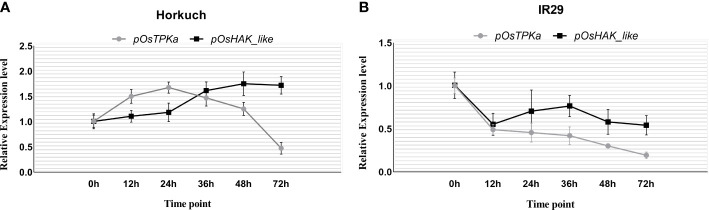
Coordinated expression analysis of OsTPKa and OsHAK_like in Horkuch **(A)** and IR29 **(B)**.

**Figure 3:**
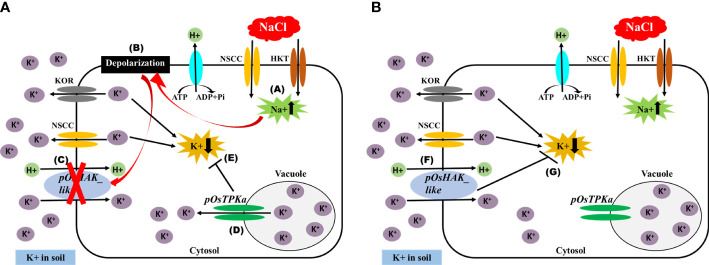
Schematic diagram of the proposed mechanism of maintaining K+/Na+ ratio in cytoplasm by coordinated function of OsTPKa and OsHAK_like. **(A)** Initially under salt stress condition, **(A)** there is influx of Na+ in cell, **(B)** which ultimately leads to membrane depolarization, **(C)** Depolarized membrane inhibits the function of HAK transporter, **(D)** Vacuolar K+ releases in cytoplasm by TPKa channel, **(E)** it helps to inhibit the decrease of K+ in cytoplasm. **(B)** at a later stage of salt stress when there is no depolarization of membrane, **(F)** HAK participates in uptaking K+ from soil and thus **(G)** helps to maintain K+ concentration in cytoplasm.

The expression of *OsTPKa* significantly increased up to 24 hours under salt compared to *OsHAK_*like, followed by a sharp reduction in the next 48 hours. In contrast, there was a gradual increase in expression of *OsHAK_like* and it was significantly higher than that of *OsTPKa* at 48 and 72 hours of salt stress, indicating that *OsTPKa* and *OsHAK_like* may work in a coordinated pattern in Horkuch to provide salt tolerance (**Figure 2A**). In IR29, no such relationship was observed (**Figure 2B**). A proposed mechanism of how the coordinated function might help Horkuch to fight saline stress has been discussed (**Figure 3**).

### 3.4 CRISPR/Cas9 mediated targeted mutagenesis of *OsTPKa* and *OsHAK_like* in the salt-tolerant variety Horkuch

The expression of *OsTPKa* and *OsHAK_like* is upregulated in tolerant Horkuch under salt-stress. Therefore, to ascertain the role of these two genes in conferring tolerance to Horkuch, a loss of function CRISPR/Cas9 mediated mutagenesis of *OsTPKa* and *OsHAK_like* was done in Horkuch. Successful transformation was achieved and is shown in **Supplementary Figures 1, 2**.

**Figure 4:**
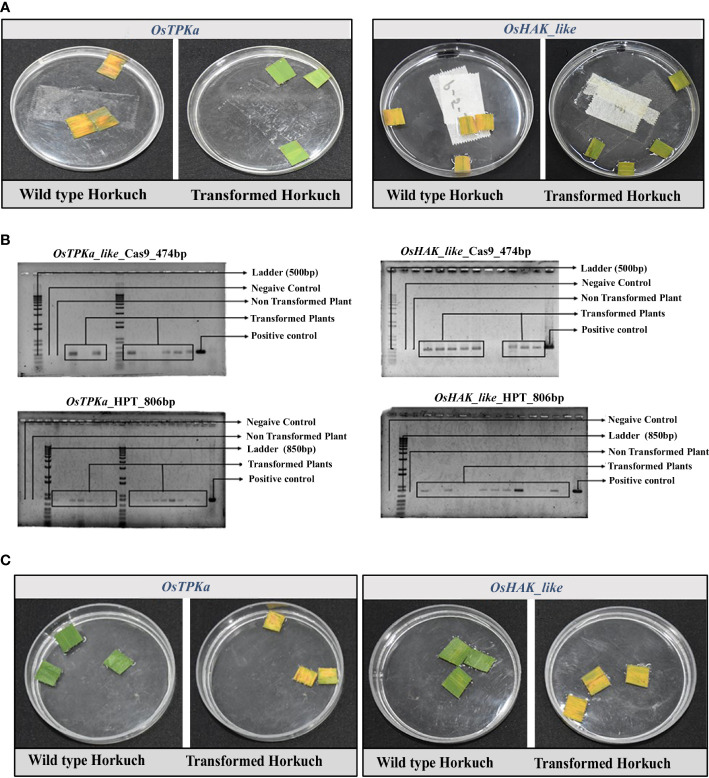
**(A)** Hygromycin resistance assay of T_0_ transformants. The wild type became yellow, but the positive transgenic lines remain green in presence of hygromycin, **(B)** molecular confirmation of transformed plants by targeting *Cas9* and *hptII* gene. The lines with the PCR bands are the positive transgenic lines, wild type showed no such bands; **(C)** Leaf disc senescence assay of flag leaves from both *OsTPKa_sgRNA* and *OsHAK_like_sgRNA* T_0_ transformants of Horkuch. sgRNA transformed transgenic lines performed poorly compared to non-transformed wild type.

Transformation was confirmed by hygromycin resistant assay which was used as the selection marker. Due to absence of hygromycin resistant gene in wild type plant, leaf pieces from the positive transformed lines remained green, whereas the wild type ones turned yellow (**Figure 4A**). The transformation rate was 64.63% and 69.83% for *OsTPKa* and *OsHAK_like* respectively. For double confirmation, we performed PCR amplification of the hygromycin positive lines using the primer sets designed to specifically amplify *Cas9* and *hptII* sequences. 77.35% and 75% of the hygromycin positive lines contained the targeted sequences for *OsTPKa* and *OsHAK_like* respectively (**Figure 4B**).

Phenotypical assessment was performed at the T_0_ stage using leaf disc senescence assay. Flag leaf pieces of non-inoculated Horkuch plants (wild type) remain mostly green after 5 days in salt solution. However, about 68% of *OsTPKa_sgRNA* and 63% of *OsHAK_like_sgRNA* transformed lines became almost yellow indicating sensitivity in salt (**Figures 4C**).

### 3.5 *OsTPKa_sgRNA* and *OsHAK_like* transformed plants showed poor phenotypical characteristics under salt-stress

**Figure 5:**
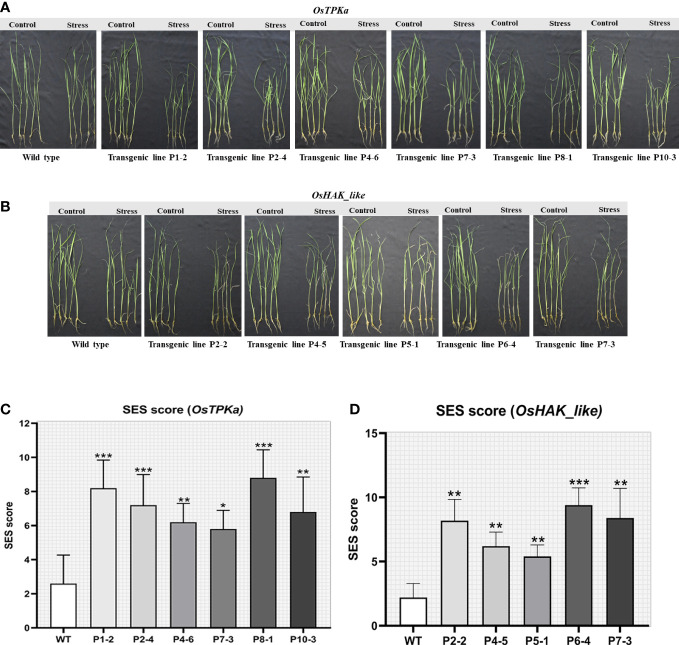
Phenotype of salt-tolerance in transformed lines. **(A, B)** The transformed and wild type seedlings growth at three leaf stage in hydroponics solution with 150 mM salt stress and no stress conditions for 9 days. **(C, D)** The comparison of seedling injury (SES score) under stress between wild type and both *OsTPKa_sgRNA* and *OsHAK_like_sgRNA* transformed plants. The stars in the graph indicates significant differences (***P < 0.001, **P < 0.01, *P < 0.1, Two way ANOVA test).

The lines which performed poorly in LDS assay were selected for the salt-stress screening at the seedling stage. The 21-day-old seedlings of transformed Horkuch and wild type were exposed to 150 mM salt-stress for *OsTPKa_sgRNA* (**Figure 5A**), where more than 70% of the transformed plants performed poorly, showing yellowing and wilting. For the *OsHAK_like_sgRNA* (**Figure 5B**) about 55% resulted in visually wilted, rolled, and yellow leaves after 9 days. The score for seedling injury (SES score) was recorded after 9 days. The transformed plants had higher SES score compared to wild type plants (**Figures 5C, D**). The lines showed other salt-sensitive phenotypes with respect to several physiological parameters.

**Figure 6:**
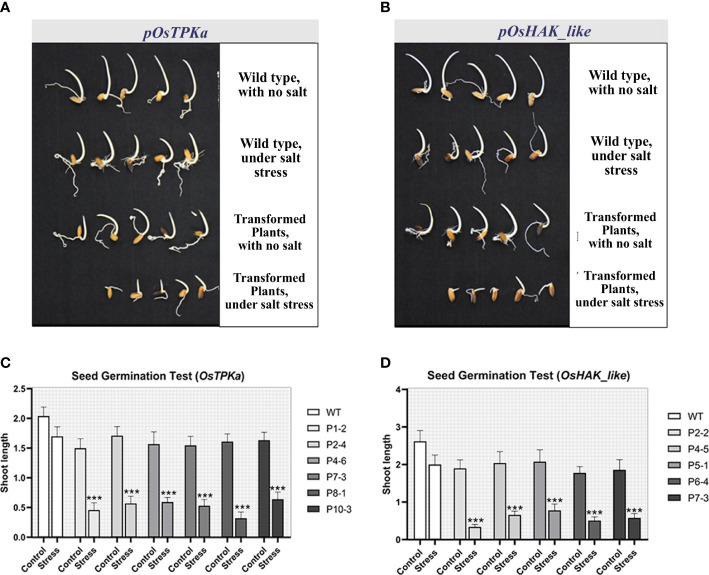
**(A, B)** Seed germination test under 100 mM salt condition. OsTPKa_sgRNA and OsHAK_like_sgRNA transformed Horkuch performed poorly under saline condition compared to their respective wild type; **(C, D)** Comparison of Shoot length of germinated seeds in seed germination test for both lines. The stars in the graph indicates significant differences (***P < 0.001, Two way ANOVA test).

After 8 days of 100 mM salt-stress, the transgenic T_1_ seeds showed higher sensitivity, poor germination rate, and significantly decreased shoot length for both *OsTPKa* (**Figures 6A, C**) and *OsHAK_like* (**Figures 6B, D**) compared to wild type indicating the loss in tolerance of transgenic Horkuch.

**Figure 7:**
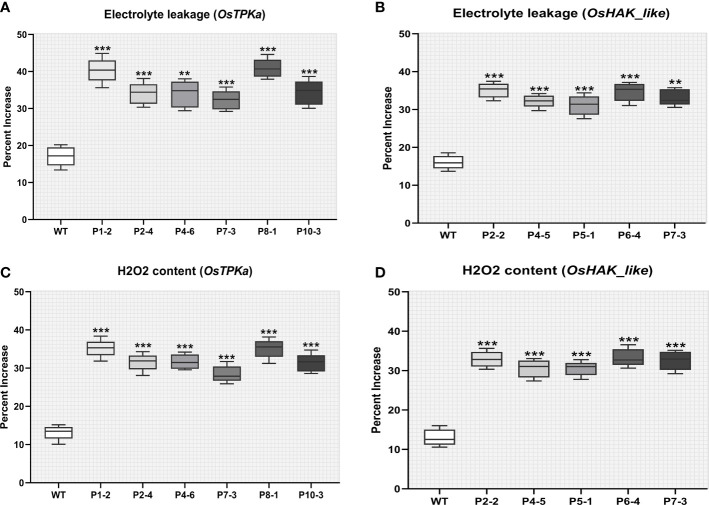
Effect of salt stress on phenotypical characteristics of transgenic plants. **(A–D)** Percentage increase in electrolyte leakage and H_2_O_2_ content; In all the parameters wild type Horkuch performed better compared to sgRNA inserted transgenic lines. The stars in the graph indicates significant differences (***P < 0.001, **P < 0.01, Ordinary one way ANOVA test).

After 9 days of salt stress, it was observed that the percent increase in both electrolyte leakage and H_2_O_2_ content was significantly higher for 6 *OsTPKa* and 5 *OsHAK_like* mutated lines compared to wild type indicating stress injury (**Figures 7A** **–D**).

**Figure 8:**
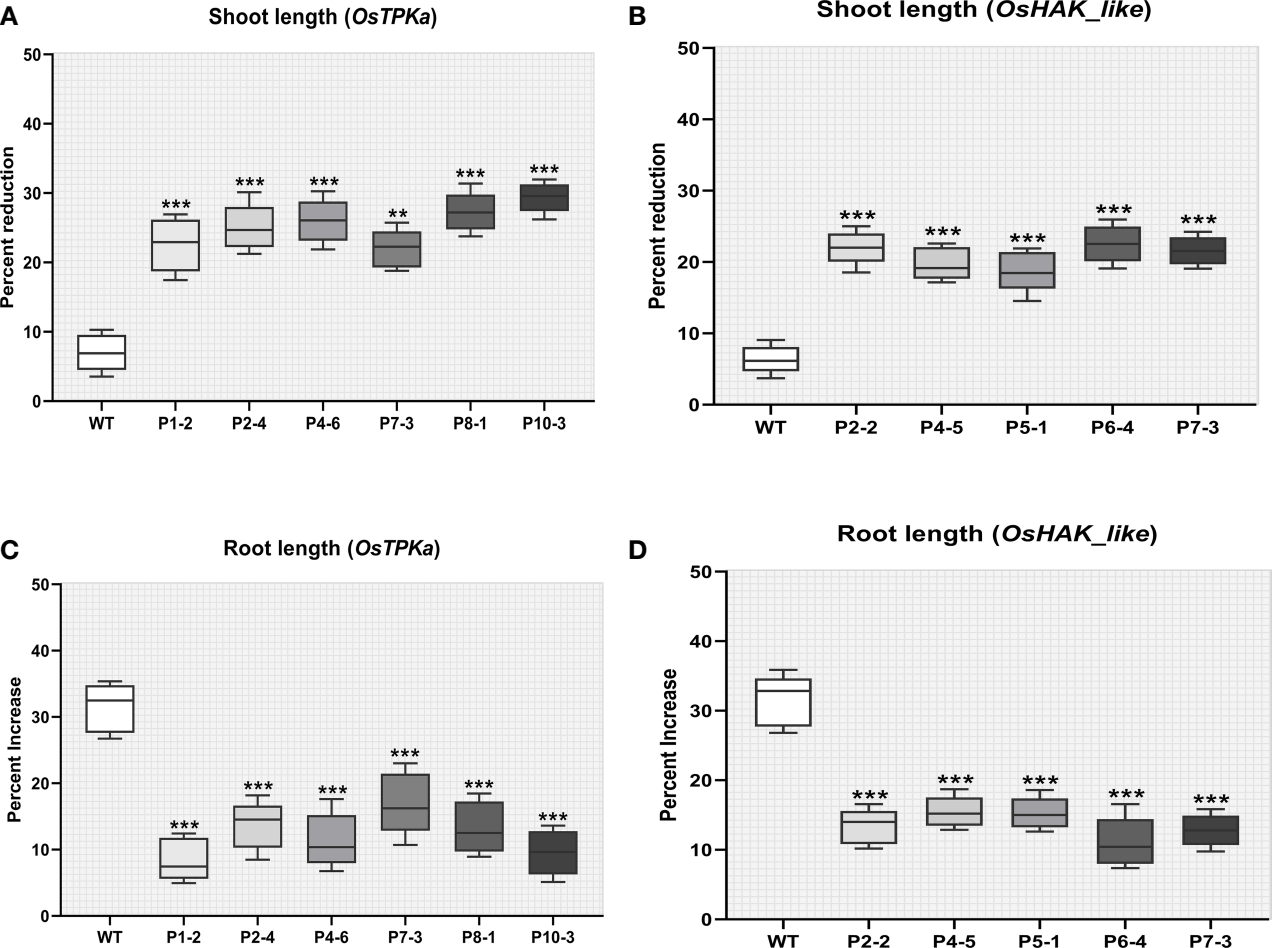
Effect of salt stress on phenotypical characteristics of transgenic plants. **(A, B)** Percentage increase in shoot length **(C, D)** Percentage reduction in root length. In all the parameters wild type Horkuch performed better compared to sgRNA inserted transgenic lines. The stars in the graph indicates significant differences (***P < 0.001, **P < 0.01, Ordinary one way ANOVA test).

The percent increase in root length and weight was significantly lower whereas the percent reduction in shoot length and weight was higher in the mutated lines compared to wild type Horkuch indicating severe salt susceptibility (**Figures 8A** **–D**).

**Figure 9:**
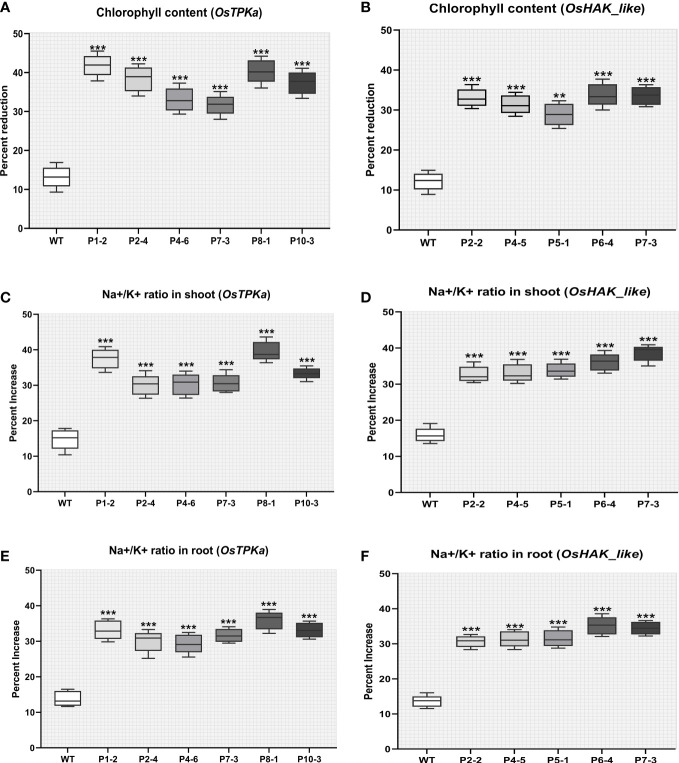
Effect of salt stress on phenotypical characteristics of transgenic plants. **(A, B)** Percent reduction in chlorophyll content; **(C, D)** Percentage increase in Na^+^/K^+^ ratio in shoot; **(E, F)** Percentage increase in Na^+^/K^+^ ratio in root under 150 mM salt condition. In all the parameters wild type Horkuch performed better compared to sgRNA inserted transgenic lines. The stars in the graph indicates significant differences (***P < 0.001, **P < 0.01, Ordinary one way ANOVA test).

The chlorophyll content was greatly reduced in the transgenic lines compared to wild type under 150 mM salt-stress indicating mutation of these genes makes a tolerant plant susceptible to chlorophyll degradation under salt stress (**Figures 9A, B**).

The percentage increase in Na^+^/K^+^ ratio was higher in transgenic lines in comparison with wild type under salt stress which indicates that the lowering of *OsTPKa* and Os*HAK_like* gene expression disrupts the cytosolic balance of Na^+^/K^+^ (**Figures 9C** **–F**)

### 3.6 Gateway technology-based overexpression of *OsTPKa* in salt-sensitive variety

*OsTPKa* gene of Horkuch was selected for cloning into the salt sensitive modern high-yielding farmer popular genotype BRRI dhan28 to test for gain of function. Molecular confirmation of the transformed plants is provided in the supplementary section (**Supplementary Figure 3**). The transformed plants showed enhanced growth and improved phenology at the T_0_ generation, compared to the wild type plants, both under 80 mM salt stress as well as without stress (**Supplementary Figure 4A**). Significant plant growth was observed in the putative transgenics in 9 days old plants but the difference between wild type and transgenics’ height reduced over subsequent days (**Supplementary Figure 4B**). In terms of effective tiller, panicle length, flag leaf length, filled grain number and filled grain weight, the transgenic plants performed better compared to the wild type plant without stress. Transgenic status was confirmed with PCR using *hpt* gene primers from the vector construct. Leaf Disc Senescence (LDS) assay from T_0_ plant flag leaves under 150 mM salt stress and PCR positive results were used to select T_1_ seeds from specific panicles for generation advancement. Leaf sections that showed greener texture under salt stress for 3 days compared to the wild type, were considered positive.

### 3.7 Overexpressed lines of *OsTPKa* performed better under salt stress

**Figure 10:**
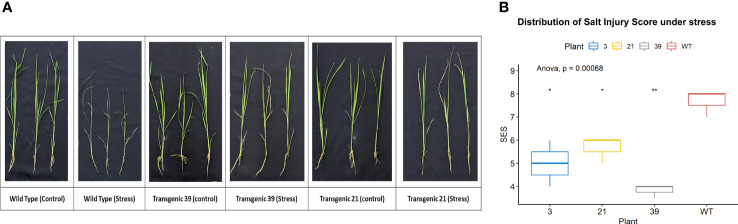
**(A)** The transgenic plants (line 39 and line 21) showed healthy appearance under 120 mM salt stress compared to the wild type plants. **(B)** Transgenic Line 39 showed significant (P<0.01) low SES score (high tolerance) than the WT. **P < 0.01, *P < 0.1.

**Figure 11:**
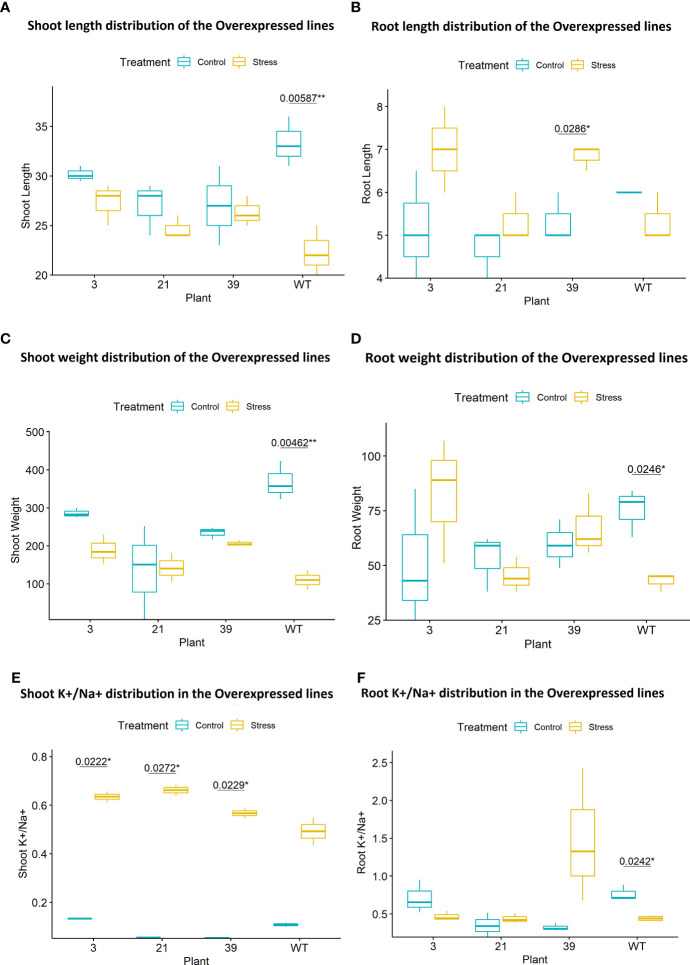
**(A)** Percent Reduction of shoot length was less in the transgenic lines and the wild type plants showed significantly high reduction (p < 0.01) under stress compared to no stress. (Transgenic lines = 3, 21, 39, WT = Wild Type BRRI dhan28). **(B)** Percent increase in root length was higher in the transgenic lines and significantly higher (p < 0.05) in line 39 under salt stress compared to no stress and the wild type plants showed reduction under stress. (Transgenic lines = 3, 21, 39, WT = Wild Type BRRI dhan28). **(C)** Percent Reduction of shoot weight was less in the transgenic lines and the wild type plants showed significantly high (p<0.01) reduction under stress compared to without stress condition. (Transgenic lines = 3, 21, 39, WT = Wild Type BRRI dhan28).**(D)** Percent reduction in root weight was less in the transgenic lines and the wild type plants under 120 mM salt stress showed significant reduction (p < 0.05) compared to without stress condition. (Transgenic lines = 3, 21, 39, WT = Wild Type BRRI dhan28). **(E)** Significant increase ( p < 0.05) in K+/Na+ ratio was observed in the shoot tissues of transgenic lines 3, 21 and 39 under salt stress compared to their without stress condition which was not observed in the wildtype. **(F)** Significant decrease (p < 0.05) in K+/Na+ ratio was observed in root in the wild type compared to no stress. Transgenic line 39 showed higher increase under salt stress. (Transgenic lines = 3, 21, 39, WT = Wild Type BRRI dhan28). **P < 0.01, *P < 0.1.

Phenotypic screening of T_1_ plants at seedling stage under 120 mM salt stress showed lower SES (salt injury), higher survival rate, lesser reduction in shoot and root length as well as shoot and root biomass (**Figures 10A** **, B**, **11A** **–D**). Leaf tissue measurements of peroxide, reduction in chlorophyll content and electrolyte leakage, were found to be less but not significantly so in the transgenic plants compared to the wild type. However, in the putative transgenic plants’ shoots, both wild type and transgenic plants showed increase in K^+^/Na^+^ ratio under salt stress but transgenic lines showed significant increase compared to no stress condition (**Figure 11** **E**). In root tissue, a significant reduction in K^+^/Na^+^ ratio was observed in the wild type plants under salt stress, which was opposite in case of the transgenic lines, specifically a higher increase was observed in line 39 (**Figure 11F**).

Relative expression analysis of the *TPKa* gene in the overexpresser T_1_ plants RNA at seedling stage also showed higher expression of the potassium channel genes in transgenic lines 3, 21 and 39 under 150 mM salt stress for 24 hr (**Supplementary Figure 5**) compared to the wild type plants. LDS assay in 3 replicates of the T_1_ plant flag leaves also confirmed better performance of the transgenic plants (**Supplementary Figure 6**). The selected transgenic plants are being advanced to T_2_ generation and will be followed up in subsequent generations for their performance.

## 4 Discussion

Rice, as a salt-sensitive cereal crop plant, experiences environmental stresses including soil salinity which ultimately affects plant growth, yield and productivity. To make matters worse, the salinity levels in the Bangladeshi coast are gradually rising due to climate change and lack of freshwater permeation due to dams in the upper riparian regions. This challenges the food security of the ever-growing population all over the world and particularly in Bangladesh. To cope with this increased soil salinity and to facilitate the development of salinity-tolerant high-yielding rice genotypes, it is imperative to understand the physiological, biochemical, and molecular events which take place in rice, especially in the tolerant varieties like- Horkuch, which are indigenous to coastal Bangladesh (Lisa et al., 2011; Razzaque et al., 2017).

The availability of contrasting genotypes of rice, like the tolerant landrace, Horkuch, and sensitive high-yielding IR29 presents a good model to dissect the cellular response towards salinity. We focused on the elucidation of differences in the mechanism of some selected salt-responsive transporters between Horkuch and IR29. From a previous study of QTL analysis between a reciprocal population derived from Horkuch and IR29 (Haque et al., 2022), the transporters *OsTPKa* (Putative vacuolar two-pore K^+^ channel) and *OsHAK_like* (Putative HAK like transporter) were targeted to elucidate their role in maintaining ionic balance under excessive Na^+^ stress.

Since both genes reside in the salt tolerance QTL from chr 3 (Haque et al., 2022), our *OsHAK_like* is from chromosome 3. However, it is different from all the 27 OsHAK transporters reported earlier (Gupta et al., 2008), including those in Chr 3. It is truncated and matches with HAK24 and 25 based on their conserved potassium transport domain spanning 56 amino acids with 72% identity (OsHAK24) and 46 amino acids with 77% identity (OsHAK25). This gene is annotated in the MSU database (LOC_Os03g55370) as well as in Uniprot (Q10EU6) and ENA (ABF99012.1) database which also shows 92% percent identity with a transporter in *Oryza barthii* (A0A0D3FPE3). Sequence based prediction with interproscan (Jones et al., 2014) suggests the role of this short protein as a putative potassium transporter. This is because it has the potassium transporter domain (interpro domain IPR003855) with two transmembrane helices and a C terminal end with hydrophilic chains residing inside (**Supplementary Figures 7, 8**). It does have the signature motif sequence **G**VVY**GK**VAMA**PLY** mentioned in Ragel et al. (2019) to be conserved as **G**VVY**GD**LGTS**PLY** (Ragel et al., 2019) where the bold amino acids are conserved for HAK members and OsHAK_like have the conserved region with lysine instead of aspartic acid, though both are hydrophilic amino acid. It is termed as hypothetical potassium transporter in the databases, but its functionality is quite evident from our results based on the seedling germination test of the transgenic plants where the gene was downregulated. On target specificity of the guideRNA for the CRISPR and primers for expression analysis was confirmed *via* genome wide blast, with no significant similarity other than our identified OsHAK_like gene on Chr 3. The separate downregulation of each of the genes, OsTPKa as well as OsHAK_like in the salt tolerant rice landrace resulted in its poorer performance. Yang et al. (2009) concluded in their study on HAK transporters that segmental duplication, tandem duplications, random translocation, and insertion events have contributed on the rice HAK family members diversity. The OsHAK_like protein under study can be an outcome of such events.

Salt tolerance has been found to be a function of a plant’s ability to maintain a higher K^+^/Na^+^ ratio in barley (Shabala and Cuin, 2008). In a study of 70 barley genotypes, it was found that K^+^ efflux was significantly correlated to salt sensitivity (Chen et al., 2007). Under salt stress, Na^+^ enters the plasma membrane through non-selective cationic channels (NSCC) as well as HKT transporters, causing strong de-polarization. This in turn activates outward rectified K^+^ channels such as KOR (Shabala et al., 2006). Simultaneously there is a rapid increase in cytosolic Ca^2+^ within seconds of exposure to NaCl (Knight et al., 1997). This in turn activates the SOS pathway, where Ca^2+^ binds SOS3 (calcium sensor protein or CBL4). SOS3 then binds and activates SOS2, a CBL interacting kinase causing phosphorylation of the plasma membrane Na^+^/H^+^ antiporter SOS1, which extrudes Na^+^ from the cell (Zhang et al., 2022). SOS2 also interacts with vacuolar H^+^ATPase, enhancing its activity which in turn drives the vacuolar Na^+^/H^+^ antiporter (NHX1) to sequester Na^+^ into the vacuole (Batelli et al., 2007). Salt stress also causes the activation of the plasma membrane H^+^ATPase by an unknown osmosensor (Shabala and Cuin, 2008). Higher H^+^ATPase activity and lowering of the cytoplasmic Na^+^ load causes the plasma membrane to re-polarize.

So how do OsTPKa and OsHAK_like, the two K^+^ transporters, which are the subject of this study fit into the salt perception and mitigation scenario? In Arabidopsis it was shown that different sets of CIPKs were activated by a Ca^2+^ signal targeting the vacuolar tonoplast transporters. As a result, the TPK channels cause efflux of K^+^ out of the vacuole to cytoplasm (Dabravolski and Isayenkov, 2022). The Ca^2+^ dependent protein kinase 3 (CDPK3) has been shown to interact with TPK1 (two-pore potassium channel homologous protein from Arabidopsis) and activate it (Latz et al., 2013). HAKs are also activated to import K^+^ across the cell into the cytoplasm. Plasma membrane CIPK-CBLs have been reported to be activated by Ca^2+^ as well as AKT1 and HAK transporters to import K^+^ from outside of the cell (Isayenkov and Maathuis, 2019).

Ability to retain the K^+^ in root and leaf tissue is an important trait for salt tolerance in plants. HAK/KUP K^+^ transporter family plays the main role for High affinity K^+^ uptake in a plants’ response to salt stress (Wu et al., 2018). Shen et al. (2015) reported involvement of OsHAK21 in the maintenance of K^+^ homeostasis. OsHAK5 overexpression improved shoot K^+^ accumulation and biomass compared to wild type plants. (Yang et al., 2014). OsHAK1-D mutated lines resulted in significantly reduced K^+^ content in shoot and root (Chen et al., 2015). These symporters need ATP for H^+^ ATPase pumping of H^+^ to maintain the H^+^ gradient (Wu et al., 2018). Moreover, the increased plasma membrane H^+^-pump activity can help K^+^ uptake *via* the HAK transporters (Shabala and Cuin, 2008).

The cytosolic K^+^ concentration needs to be restored after Na^+^ induced signaling and membrane depolarization for continuing normal cellular metabolism. The balance between K^+^ efflux and K^+^ uptake determines tolerance to salt stress. These 2 processes are maintained by K^+^ channels and transporters regulated in a cell and tissue specific manner. K^+^ efflux is mainly mediated by GORK (guard cell outward rectifying K^+^ channel) type and ROS-activated NSCC-type channels (Jayakannan et al., 2013; Wu et al., 2015). Wu et al. (2018) suggested a probable coordination between TPK, TPC channels and 14-3-3 proteins, where increased expression of the latter activated the TPK but inactivated the TPC channels resulting in replenishing of the cytosolic K^+^ from vacuolar pool. TPK channels can act as osmosensor in case of high external osmotic pressure, since they are osmo sensitive (MacRobbie, 2006). Hence activation of TPK channels ensures rapid release of K^+^ from vacuole which is the main cellular depository of osmotica and water (Maathuis, 2011).

### 4.1 *In silico* analysis of *OsTPKa* and *OsHAK_like* between Horkuch and IR29 showed significant differences in stress-responsive motifs in promoter region

In the study of *In silico* promoter analysis, for both *OsTPKa* and *OsHAK_like*, there were significant differences in stress-related cis regulatory element in promoter region such as- presence of higher number of Auxin, Jasmonate, Gibberellin, Abscisic acid responsive and dehydration responsive motifs in Horkuch. Phytohormones like auxin, jasmonate, gibberellin, abscisic acid regulates plant growth adaptation under salt stress. Additionally, these hormones help plant build a defense system to fight against stress (Yu et al., 2020). Therefore, the presence of these motifs in higher number might account for the greater responsiveness of those genes to salt-stress stimuli in the tolerant rice landrace.

### 4.2 Tolerant genotypes might get survival advantage due to increased expression of *OsTPKa* and *OsHAK_like* transporter under salt-stress

In the tissue specific expression analysis study (**Figure 1**) and salt-responsive genotype specific expression analysis study (**Figure 2**), *OsTPKa* and *OsHAK_like*, a hypothetical protein of HAK (High affinity K^+^ transporter) family were found to be upregulated in roots of the salt-tolerant Horkuch, Pokkali and I-14 compared to sensitive IR29, indicating that the increased expression of these 2 genes is perhaps important for the performance of tolerant varieties under saline conditions. During the onset of salt stress, the rapid increase in Ca^2+^ may lead to compensatory K^+^ efflux into cytoplasm from the vacuole through activation of TPK channels to replenish stress-induced cytosolic K^+^ depletion (Latz et al., 2007). So, the ideal scenario is to increase K^+^ selective TPK activity (Wu et al., 2018). Again, HAK is the only K^+^ uptake transporter that is known to operate in K^+^ uptake from soil at external concentrations well below 10 µM and this characteristic activity for high-affinity K^+^ transport renders plants expressing the HAK transporter genes very tolerant to low-K^+^ conditions (Assaha et al., 2017). Our hypothesis is strengthened by the fact that the separate downregulation of each of the genes, Os*TPKa* as well as *OsHAK_like* in the salt tolerant rice landrace led to poorer seed germination both without and with salt. The reduction in shoot length was statistically significant (**Figures 6A-D**). Therefore, upregulation of *OsHAK_like* in the tolerant varieties likely mediates the higher uptake of K^+^ from soil during salt-stress.

### 4.3 *OsTPKa* and *OsHAK_like* might work in a coordinated manner to combat salinity stress induced K^+^ depletion in cytoplasm

From the coordinated expression analysis study of *OsTPKa* and *OsHAK_like* (**Figures 2**, **3**) in root of Horkuch under salt-stress it was found that there was a slow but gradual increase in expression of *OsHAK_like* whereas for *OsTPKa*, the expression was higher at the initial stage and decreased at the latter stages of salinity stress. It is possible that at the initial stage of salinity stress, the vacuolar TPKa is activated *via* the rapid increase in Ca^2+^ (Latz et al., 2007) and the rapid de-polarization of the membrane and K^+^ efflux is countered. Meanwhile, the released Ca^2+^ reduces the Na^+^ load by activating the SOS pathway, while the simultaneous H^+^ pump activity further re-polarizes the membrane which in turn may subsequently activate the HAK_like K^+^ transporter (Shabala and Cuin, 2008). Therefore, tolerant plants may rely on a rapid replenishment of K^+^ supply from vacuolar storage *via* TPK mediated K^+^ efflux into the cytoplasm to maintain osmotic balance and to normalize membrane potential. The activity of the TPK may allow the cells the time to complete the signaling processes and increase the number of transcripts for high affinity transporters (HAK) to further increase K^+^ uptake and restore the cellular K^+^ pool volume (Wu et al., 2018). Now, when HAK transporters take part in maintaining K^+^ in cytoplasm, there is no need for vacuolar storage support and finally, other vacuolar inward transporters help replenish the lost K^+^ in the vacuole. Therefore, from this study, it can be hypothesized that both genes may work in a coordinated manner to increase the chance of survival under salinity stress in Horkuch. However, this hypothesis needs to be proven with by evaluation of the role of both potassium and Ca^2+^ in this coordination which are being planned.

### 4.4 *OsTPKa* and *OsHAK_like* mutant transgenic lines showed a decrease in tolerance suggesting their role in survival under salinity

The expression analysis data of *OsTPKa* and *OsHAK_like* in the roots showed the overexpression of both genes in tolerant varieties like Horkuch and Pokkali compared to the sensitive one (IR29) under salt stress. However, the mechanism of how the upregulation of these two genes helps maintain a favorable ion gradient in tolerant varieties is not well characterized yet. So, to gain insight into the biophysical properties and physiological function of these genes, they were subjected to CRISPR/Cas9-based loss of function mutation in Horkuch.

It was observed that even at the T_0_ generation, the mutant lines of Horkuch showed a remarkable sensitivity to 150 mM NaCl in leaf disc senescence assay indicating loss of tolerance in the transformed lines. Among selected lines that showed a sensitive response, 6 lines each for *OsTPKa* and 5 for *OsHAK_like* performed poorly with an extremely slower rate of seed germination, a significant reduction in shoot length and weight, and a very low percent increase in root length compared to wild type. Salinity-induced osmotic and ionic stresses are known to inhibit and delay seed germination along with interruption of nutrient uptake and growth retardation, especially and more prominently in sensitive varieties (Hakim et al., 2010). Therefore, our study indicates that due to mutation of the above-mentioned genes, tolerant Horkuch is behaving more like a sensitive variety which points to the importance of the two transporter genes in providing salinity tolerance.

To further verify the reduction in stress-tolerant characteristics of T_1_ Horkuch *OsTPKa* and *OsHAK_like*, mutant lines, various tests including electrolyte leakage, H_2_O_2_ concentration and chlorophyll content were measured. Electrolyte leakage and H_2_O_2_ in shoots showed an increase with stress in the Horkuch mutant lines compared to the wild type indicating disturbed membrane integrity (Mishra et al., 2021). Percent increase in electrolyte leakage and peroxide (H_2_O_2_) content due to salt stress was lesser in the overexpressed lines under salinity compared to the wild type plants. For the mutant line, the content of the photosynthetic pigment in rice plant grown under salt condition declined significantly, being especially susceptible to salt. The chlorophyll pigment content in leaves of tolerant rice genotypes under stress is known to be maintained better than in sensitive varieties. In our study, the reduction of chlorophyll content was significantly higher in the mutant Horkuch lines which can lead to a decrease in photosynthetic efficiency and sugar content and a subsequently negative effect on plant survival under stress (Amirjani, 2011). There was striking increase in Na^+^/K^+^ ratio in *OsTPKa* and *OsHAK_like* downregulated lines compared to corresponding wild type which indicates the importance of above-mentioned genes in maintaining the appropriate K^+^ concentration under salt stress.

### 4.5 *OsTPKa* overexpressed transgenic lines showed increased tolerance to salinity

The overexpressed lines where the *OsTPKa* gene from Horkuch was cloned and transformed in a salt sensitive genotype BRRI dhan28, showed a significant increase in growth during germination as well as during initial growth at seedling stage. They looked healthy and taller than their wildtype counterpart even without any stress application (**Supplementary Figure 4A**). However, this significant difference with the wild type plants were observed only during the earlier growth of the plants. At 12 days old, wild type plants and putative transgenic plants were of similar height. Differences became evident between WT and transgenic plants, where the latter showed significantly lower reduction in plant height after salinity stress of 18 days (**Supplementary Figure 4B**). The putative transgenic plants’ survival rate was much higher under salt stress at the seedling stage. This interesting observation indicates that the overexpressed lines may have some kind of growth advantage due to supply of extra potassium during the early days compared to the wild type even without salt stress. The plants without stress were subjected to generation advancement and a panicle wise selection using LDS assay could select the lines for phenotypic screening at seedling stage in the T_1_ generation.

Under salt stress, in the over-expressed lines of *OsTPKa*, the root length and weight were significantly higher and there was significant increase of K^+^/Na^+^ under salt stress in the transgenic lines compared to the wild type plants. They showed less percent reduction of chlorophyll content under salt stress compared to the wildtype plant. However, this change was not that significant indicating that the overexpressed lines get growth advantage for their survival and maintenance but may not have extra benefit for enhanced photosynthesis or chlorophyll production in the leaf tissues.

The overexpressed T_1_ transgenic lines showed significantly higher K^+^/Na^+^ ratio in their shoot and root tissues compared to no stress condition. In the wild type plants’ root tissue, a significant reduction was observed under salt stress as opposed to the transgenic plants. The root tissues of transgenic line 39 showed a highly significant rise in K^+^/Na^+^ content compared to their control conditions, while the wild type plant showed a significant reduction. This trend was also supported by the higher gene expression level of OsTPKa in root tissue of line 39 compared to the other lines (**Supplementary Figure 5**). Potassium is one of the major nutrients required for growth and development of plants. Not only does early vigor give the plant better survivability under salt stress, but the higher potassium also protects against the toxic effects of the sodium. Therefore, the study supports the possible role of *OsTPKa* and *OsHAK_like* in providing salt tolerance.

## 5 Conclusion

CRISPR-mediated knockout of the two transporter genes, *OsTPKa* and *OsHAK_like* in salt-tolerant genotype (Horkuch) made it very sensitive to salt stress. The transformed plants showed poorer performance in several phenological tests. Moreover, *OsTPKa* from Horkuch when cloned and transformed into sensitive genotype, BRRI dhan28, conferred salt tolerance to the latter. The loss and gain of function study indicated the important role of the OsTPKa channel and OsHAK_like transporter in conferring salt tolerance in the rice plant. Collectively, this study helped us understand that the time-specific increased activity of these two transporters may help the rice landrace Horkuch defend against salt stress. It also is indicative of the likely mechanism by which this rice landrace has favorably adapted to coastal salinity over many years. The loci of these two transporters are close enough (Haque et al., 2022) for these to be simultaneously bred into commercial rice genotypes by use of DNA markers. Alternatively, the simultaneous activation of their respective promoters through gene-editing may lead to the quick development of salt tolerant high-yielding rice.

## Data availability statement

The original contributions presented in the study are included in the article/**Supplementary Material**. Further inquiries can be directed to the corresponding author.

## Author contributions

UH, SE and IJ performed the lab works and screening. UH, SE and ZS wrote the manuscript. ZS and SE designed the experiment. All authors contributed to the article and approved the submitted version.
