# Supplementary material for: Functional genomic analysis of K+ related salt-responsive transporters in tolerant and sensitive genotypes of rice
Source: Front Plant Sci. 2023 Jan 19;13:1089109. doi: 10.3389/fpls.2022.1089109 (PMC9893783; doi:10.3389/fpls.2022.1089109)
Supplement: Supplementary file 1 [file DataSheet_1.pdf]

Supplementary Data:

Supplementary Table 1: List of Primers used for qRT PCR, PCR confirmation and sequencing.

List of primers used in quantitative Real-Time gene expression analysis				
Name of the genes	**Primer set**	**Name and Sequence of Primers**	**Annealing temperature (°C)**	**Product size (bp)**
eEF-1α	Primer set 1	eEF-1α_FP<br>5´-TTTCACTCTTGGTGTGAAGCAGAT-3	62.7	103
		eEF-1α_RP<br>5´-GACTTCCTTCACGATTTCATCGTAA-3´		
OsTPKa	Primer set 2	*OsTPKa*_RT_FP<br>5'-AGACCTCGTCCCAAACAATG-3'	62.3	173
		*OsTPKa*_RT_RP<br>5'-TCTTGGTCTCACCACCCTTC-3'		
OsHAK_like	Primer set 3	*OsHAK_like*_RT_FP<br>5'-AGACGCCAAACTTGGTCAAA-3'	62.1	194
		*OsHAK_like*_RT_RP<br>5'-GGCTCTGGTACCCAATTGAA-3'		
List of primers used for PCR confirmation of sgRNAs cloned plasmids				
Name of the genes	**Primer set**	**Name and Sequence of Primers**	**Annealing temperature (°C)**	**Product size (bp)**
OsTPKa	Primer set 4	*OsTPKa*_sgRNA_confirm_FP_1<br>5'-GGCAGCTTTTATCCCCATAACCCA -3'	64°C	850
		*OsTPKa*_sgRNA_confirm_RP_1<br>5´-GCTGATGCGAGGAGAGGAGATGAGG-3´		
	Primer set 5	*OsTPKa*_sgRNA_confirm_FP_2<br>5´-CACTCATTAGGCACCCCAGG-3´	61.1°C	521
		*OsTPKa*_sgRNA_confirm_RP_2<br>5'-AAACTGGGTTATGGGGATAAAAGC -3'		
OsHAK_like	Primer set 6	*OsHAK_like*_sgRNA_confirm_FP_1<br>5'-GGCAGTTCGGACGGGATGATACCG-3'	66°C	850
		*OsHAK_like*_sgRNA_confirm_RP_1		

		5´-GCTGATGCGAGGAGAGGAGATGAGG-3´		
	Primer set 7	*OsHAK_like*_sgRNA_confirm_FP_2<br>5´-CACTCATTAGGCACCCCAGG-3´	62.5°C	521
		*OsHAK_like*_sgRNA_confirm_RP_2<br>5'-AAACCGGTATCATCCCGTCCGAAC-3'		
List of primers for sequencing of sgRNA cloned plasmids				
Name of the genes	**Primer set**	**Name and Sequence of Primers**	**Annealing temperature (°C)**	**Product size (bp)**
OsTPKa, *OsHAK_like*	Primer set 8	sgRNA_sequencing_FP<br>5´-CAGCGTAGTACTGCAGCCCA -3´	66°C	401
		sgRNA_sequencing_RP<br>5´-AGTCCAAACCACAACCCGC -3´		
List of Primers used for the amplification of the desired genes for the Directional pENTR/D-TOPO cloning reaction				
Name of the genes	**Primer set**	**Name and Sequence of Primers**	**Annealing temperature (°C)**	**Product size (bp)**
OsTPKa	Primer set 9	*OsTPKa*_clone_FP<br>5´-CACCATGGATGACAACAGCATTCA-3´	61	1044
		*OsTPKa*_clone_RP<br>5´-TCACTGAGCAGATTGTGCTAGG-3´		
List of primers used for PCR confirmation of *OsTPKa* gene cloned in *pENTR* vector				
Name of the genes	**Primer set**	**Name and Sequence of Primers**	**Annealing temperature (°C)**	**Product size (bp)**
OsTPKa	Primer set 10	M13 (-20)_FP<br>5´-TGTAAAACGACGGCCAGT-3´	62	665
		*OsTPKa*_clone_RP<br>5´-TCACTGAGCAGATTGTGCTAGG-3´		
		*OsTPKa*_clone_FP	61	1044

	Primer set 11	5´-CACCATGGATGACAACAGCATTCA-3´		
		*OsTPKa*_clone_RP<br>5´-TCACTGAGCAGATTGTGCTAGG-3´		
List of primers for sequencing of the *OsTPKa* cloned plasmids				
Name of the genes	**Primer set**	**Name and Sequence of Primers**	**Annealing temperatur e (°C)**	**Product size (bp)**
OsTPKa	Primer set 12	M13 (-20)_FP<br>5´-TGTAAAACGACGGCCAGT-3´	62	
		M13_RP<br>5´-CAGGAAACAGCTATGAC-3´		

Supplementary Table 2: Potential candidate gRNA specific for *OsTPKa* and *OsHAK_like* gene disruption

**Gene**	**Guide sequence**	**Location**	**GC content**	**On score**	**Off score**
OsTPKa	GCTTTTATCCCCATAACCCAGGG	CDS	45%	0.9209	13
OsHAK_like	GTTCGGACGGGATGATACCGCGG	CDS	60%	0.8726	23

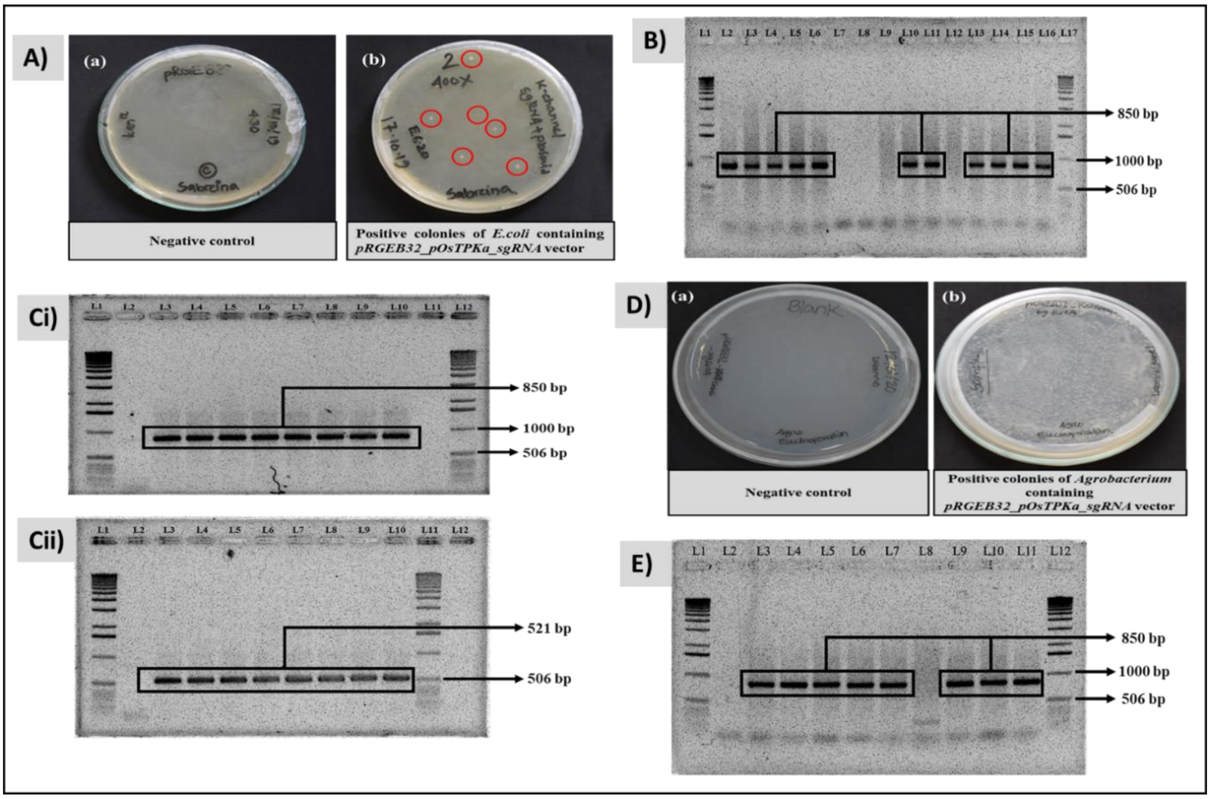

Supplementary Figure 1: Construction and transformation of *OsTPKa_sgRNA*. A) Transformation of *pRGEB32_OsTPKa_sgRNA* vector into *E. coli*. B) Confirmation of *pRGEB32_OsTPKa_sgRNA* vector in *E. coli* by lysate PCR. Ci) Confirmation of the insertion of the forward spacer sequence of sgRNA into the vector *pRGEB32;* Cii) Confirmation of the insertion of the reverse spacer sequence of sgRNA into the vector *pRGEB32*, D) Transformation of *pRGEB32_OsTPKa_sgRNA* vector into *Agrobacterium*, E) Confirmation of *pRGEB32_OsTPKa_sgRNA* vector into *Agrobacterium.*

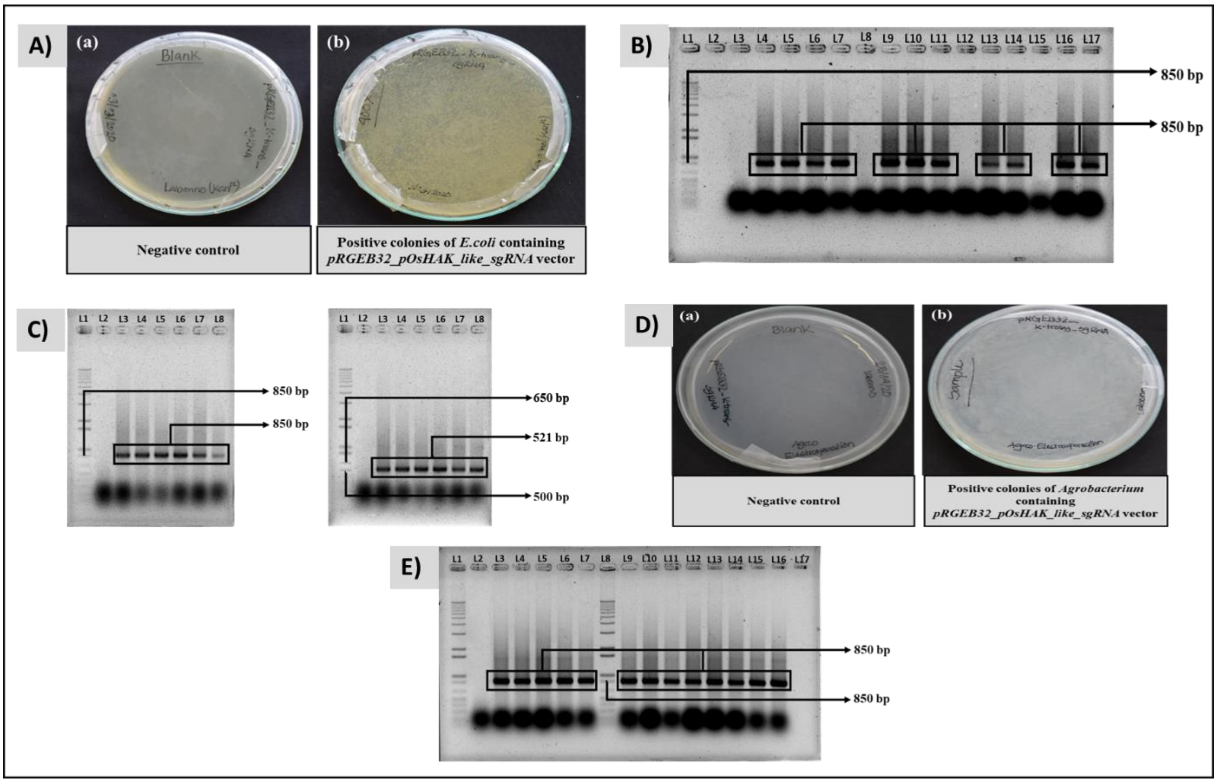

Supplementary Figure 2: Construction and transformation of *OsHAK_like_sgRNA*. A) Transformation of *pRGEB32_OsHAK_like_sgRNA* vector into *E. coli*. B) Confirmation of *pRGEB32_OsHAK_like_sgRNA* vector in *E. coli* by lysate PCR. Ci) Confirmation of the insertion of the forward spacer sequence of sgRNA into the vector *pRGEB32;* Cii) Confirmation of the insertion of the reverse spacer sequence of sgRNA into the vector *pRGEB32*, D) Transformation of *pRGEB32_OsHAK_like_sgRNA* vector into *Agrobacterium*, E) Confirmation of *pRGEB32_OsHAK_like_sgRNA* vector into *Agrobacterium.*

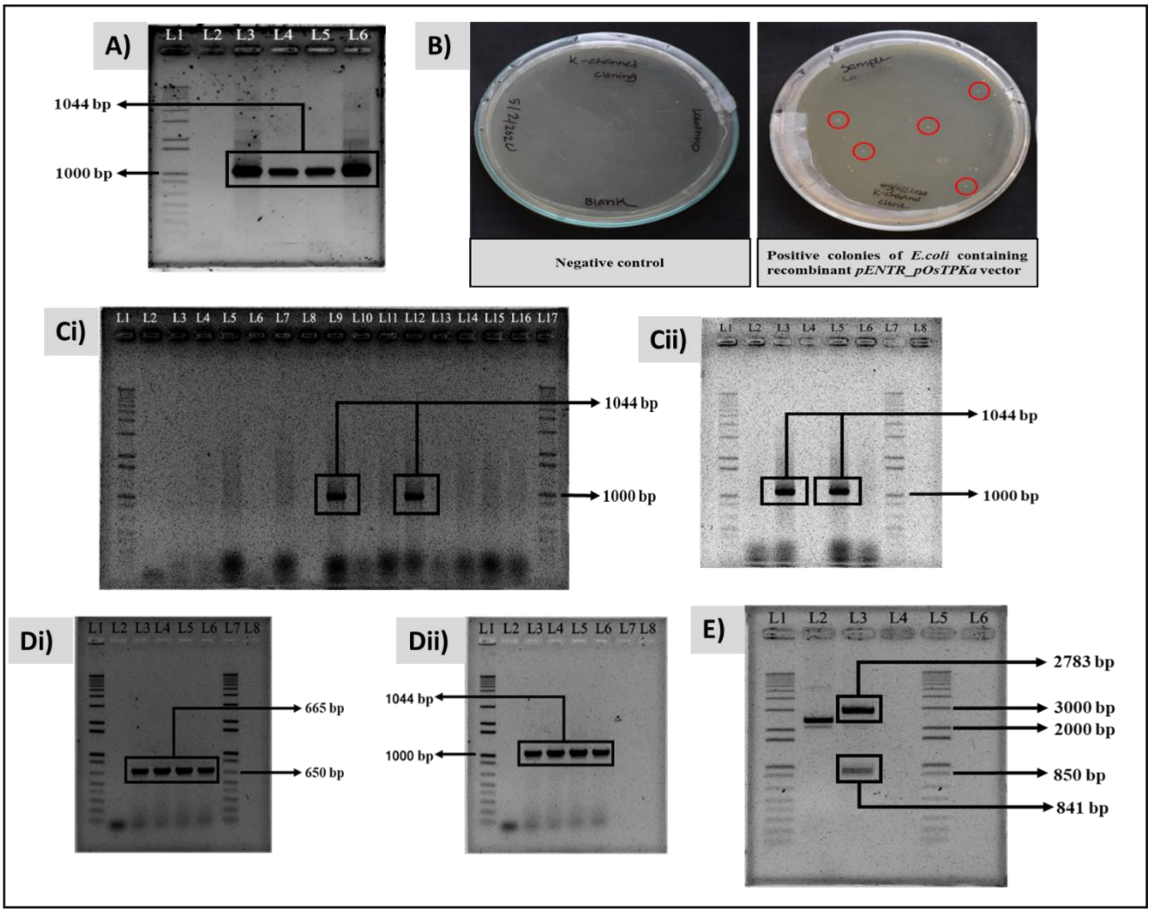

Supplementary Figure 3: A) PCR amplification of *OsTPKa* cDNA from Horkuch, B) Transformation of *pENTR_OsTPKa* vector into *E. coli*, C) Confirmation of *pENTR_OsTPKa* vector in *E. coli* by lysate PCR, D) Confirmation of *pENTR_OsTPKa* vector through PCR analysis of isolated raw plasmid i) by M13 forward and internal reverse primer of gene, ii) by forward and reverse primer of *OsTPKa*. E) Restriction digestion confirmation of cloning with *PstI* enzyme.

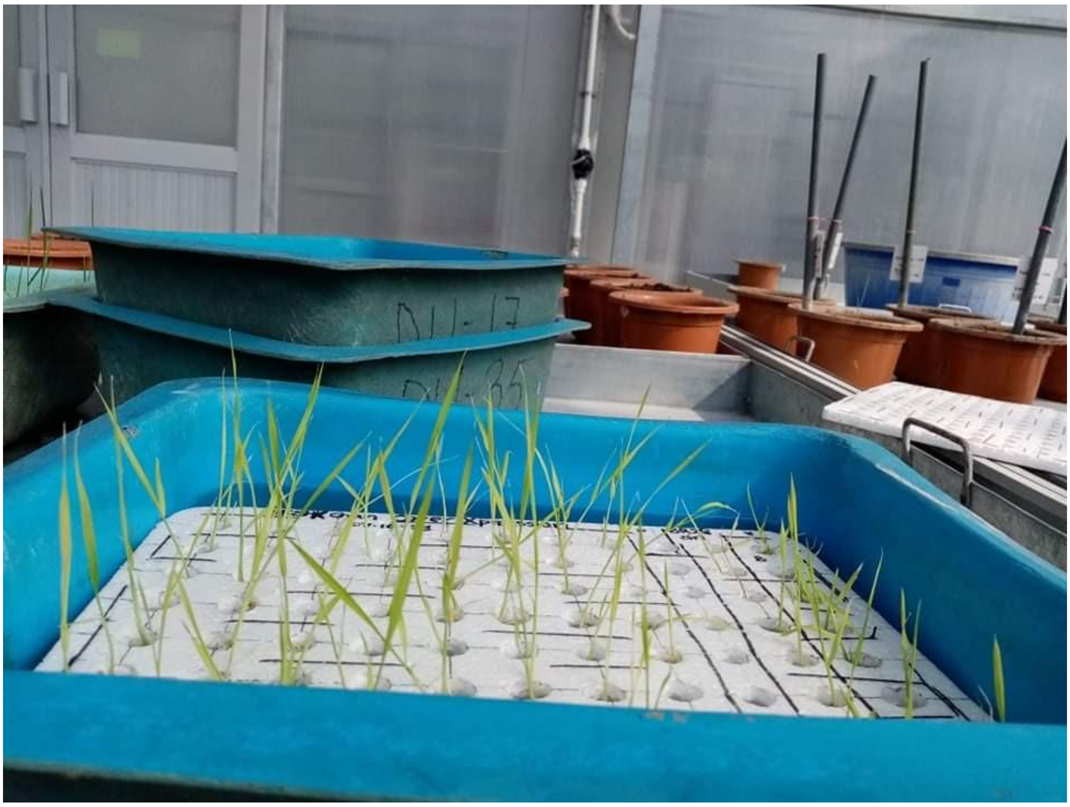

Supplementary Figure 4 (a): The 9 day old plants in the hydroponic solution showing difference between the putative transgenic plants (left side) and the wild type plants (right most 2 lanes)

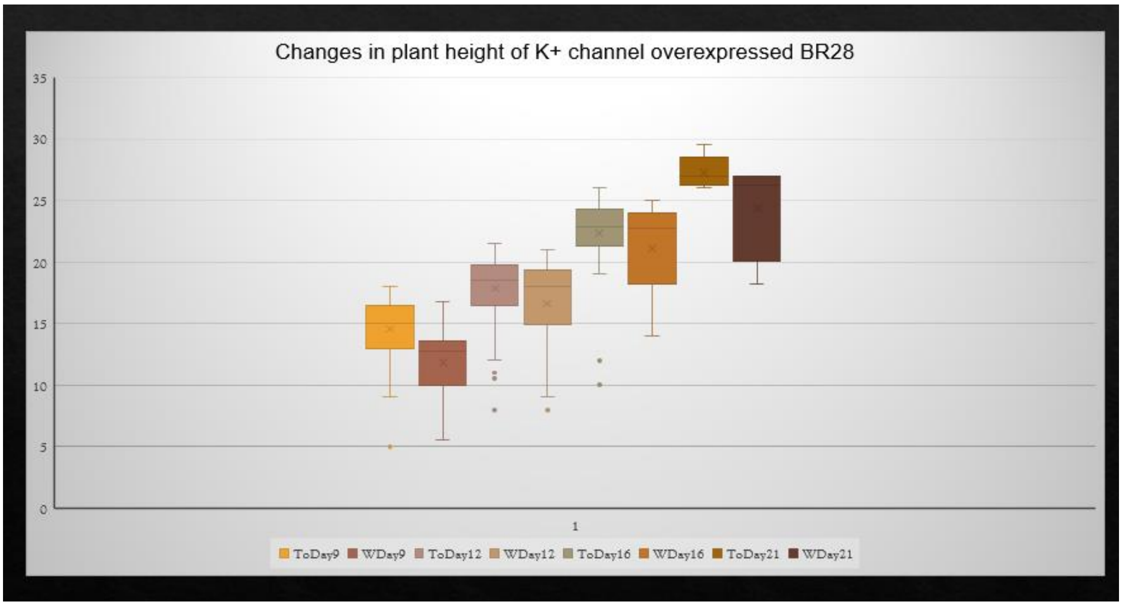

Supplementary Figure 4 (b): The 9 day old plants showed growth difference between putative transgenics and the wildtypes but the difference was less prominent afterwards

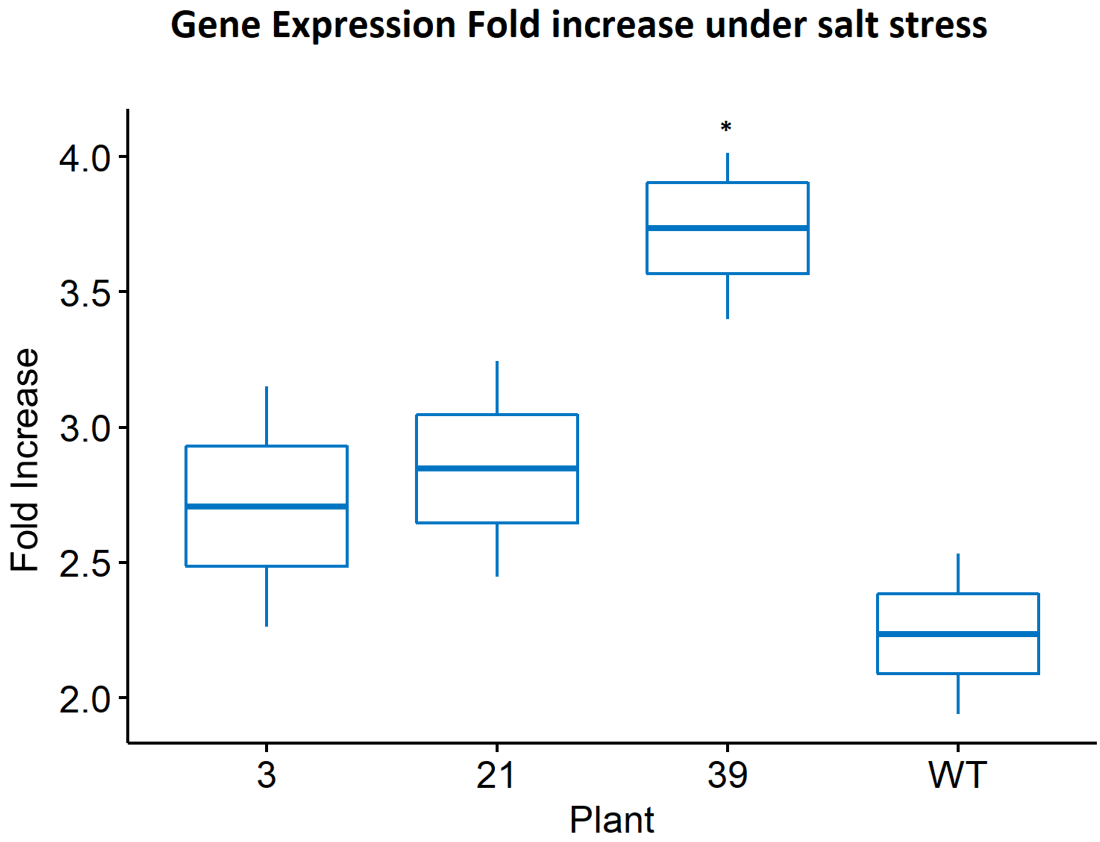

Supplementary Fig 5: Relative gene expression analysis using qRT-PCR of the potassium channel gene showed higher gene expression level in the transgenic lines 3, 21 and 39 (* = p<0.1) compared to the wild type under 150mM salt stress. (Transgenic lines = 3, 21, 39, WT = Wild Type BRRI dhan28)

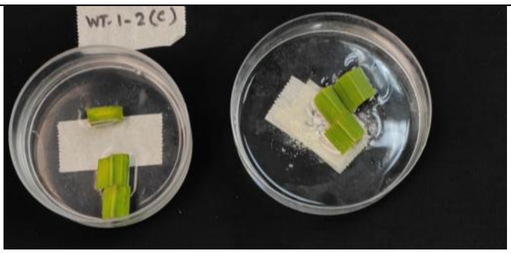	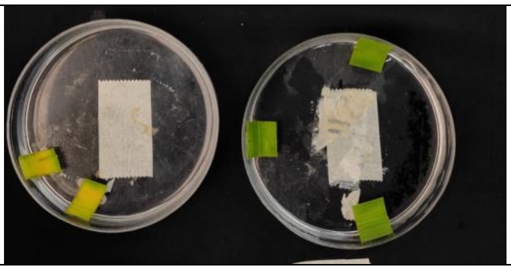
**Under No Stress (Left: Wild Type, Right : Transgenic line 21)**	**Under Salt Stress (Left: Wild Type, Right : Transgenic line 21)**

Supplementary Figure 6: Leaf Disc assay of flag leaves of specific T_1 plants showed healthier appearance under 150mM salt stress compared to the wildtype

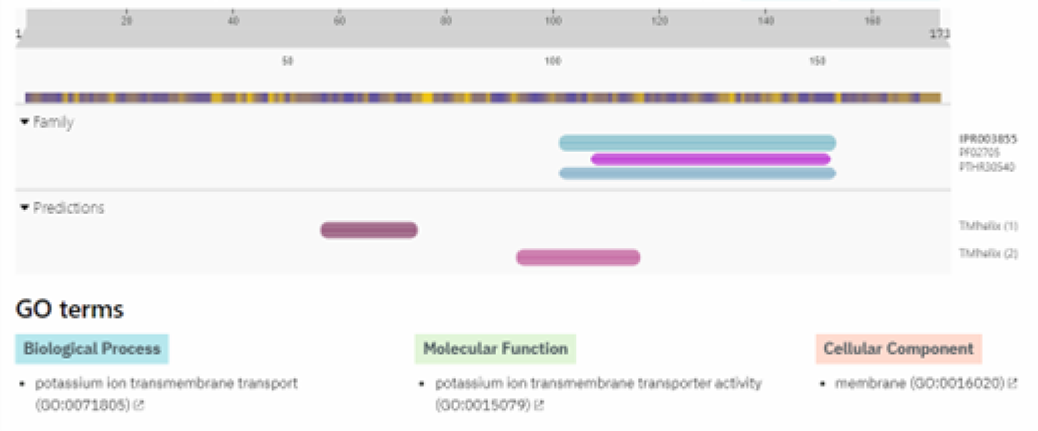

Supplementary Figure 7: Interpro domain result showing the existence of the full potassium transport domain in the hypothetical protein

```
# WEBSEQUENCE Length: 173
# WEBSEQUENCE Number of predicted TMHs:  2
# WEBSEQUENCE Exp number of AAs in TMHs: 37.0385
# WEBSEQUENCE Exp number, first 60 AAs:  10.82254
# WEBSEQUENCE Total prob of N-in:        0.71567
# WEBSEQUENCE POSSIBLE N-term signal sequence
WEBSEQUENCE     TMHMM2.0        inside       1    56
WEBSEQUENCE     TMHMM2.0        TMhelix     57    74
WEBSEQUENCE     TMHMM2.0        outside     75    93
WEBSEQUENCE     TMHMM2.0        TMhelix     94   116
WEBSEQUENCE     TMHMM2.0        inside     117   173
```

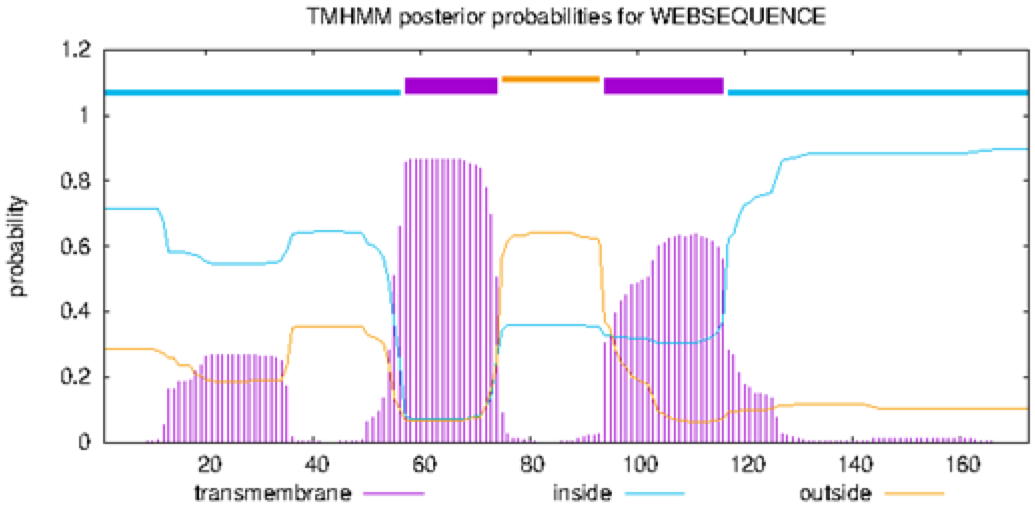

Supplementary Figure 8: TMHMM analysis result showing the existence of two trans membrane helices and hydrophilic c terminal tail in the hypothetical potassium transporter
